# Cost-effectiveness, pharmaceutical pricing and the value flower – doing some gardening

**DOI:** 10.1007/s11019-026-10373-z

**Published:** 2026-07-07

**Authors:** Lars Sandman, Martin Henriksson, Erik Gustavsson, David Lindqvist, Niklas Juth

**Affiliations:** 1 Department of health, medicine and caring sciences, National Centre for Priorities in Health, Linköping university, Hus 511, ingång 76 eller 78, plan 13, Campus US, Lasarettsbacken, 581 83 Linköping, Sweden; 2 Division of Philosophy and Applied Ethics, Department of Culture and Society, Linköping University, Campus Valla, Key, Ingång Key, 581 83 Linköping, Sweden; 3 Institute for Futures Studies, Stockholm, Sweden; 4 Department of Public Health and Caring Sciences, Centre for Research and Bioethics, Uppsala university, Husargatan 3, 752 37 Uppsala, Sweden

**Keywords:** Cost-effectiveness, Just pricing, Health economy, Health technology assessment, Pharmaceuticals, Value theory

## Abstract

During the recent years, ISPOR has proposed a number of values that could be taken into account in health technology assessments, for instance when doing an evaluation of what determines society’s price for pharmaceutical treatments. These includes values that are not usually considered in health economic evaluation of pharmaceutical pricing, for instance option value, the value of hope and knowing, insurance value, the value of avoiding contagion, and scientific spillover. If these values were included in assessments of pricing of pharmaceutical, it would lead to broadening the cost-effectiveness analysis. We scrutinize the these suggested new value petals of the so-called value flower from two points of views: ethical value theory and the practical implications for a public healthcare system of a European kind, which factors in considerations of traditional cost-effectiveness (primarily in terms of net cost and quality-adjusted life years) and justice (primarily in terms of severity) from the outset. We argue that some of the suggested value petals should not be included in the health economic assessment for several reasons related to value theory or practical considerations in the welfare state: some are mutually conflicting, some do not point out unequivocally positive values, some entails double counting, some are already included in healthcare systems with universal coverage, or can be better handled by other means than by affecting pharmaceutical pricing. A few new petals have potential merit, for instance equity, but much work, both normative and empirical, is required if they should be included in the assessment of pharmaceuticals.

## Introduction

Cost-effectiveness evidence plays an important role in the allocation of scarce resources in publicly funded health care systems, most explicitly formalized in decision making concerning the reimbursement of pharmaceuticals. The cost-effectiveness evidence is generated by economic evaluations that typically assess the ´value´ of new pharmaceuticals by comparing the costs and health outcomes of two or more interventions. In several countries these values are important input to reimbursement decisions that determine the price of pharmaceuticals, often together with justice-based or equity considerations such as giving priority to patients with more severe diseases (Barra et al. 2020). From an ethical perspective it seems obvious that how we distribute scarce resources in healthcare is a matter of values and that we should distribute resources, including pharmaceuticals, in a way that reflects what we value in and with healthcare.

**Fig. 1:**
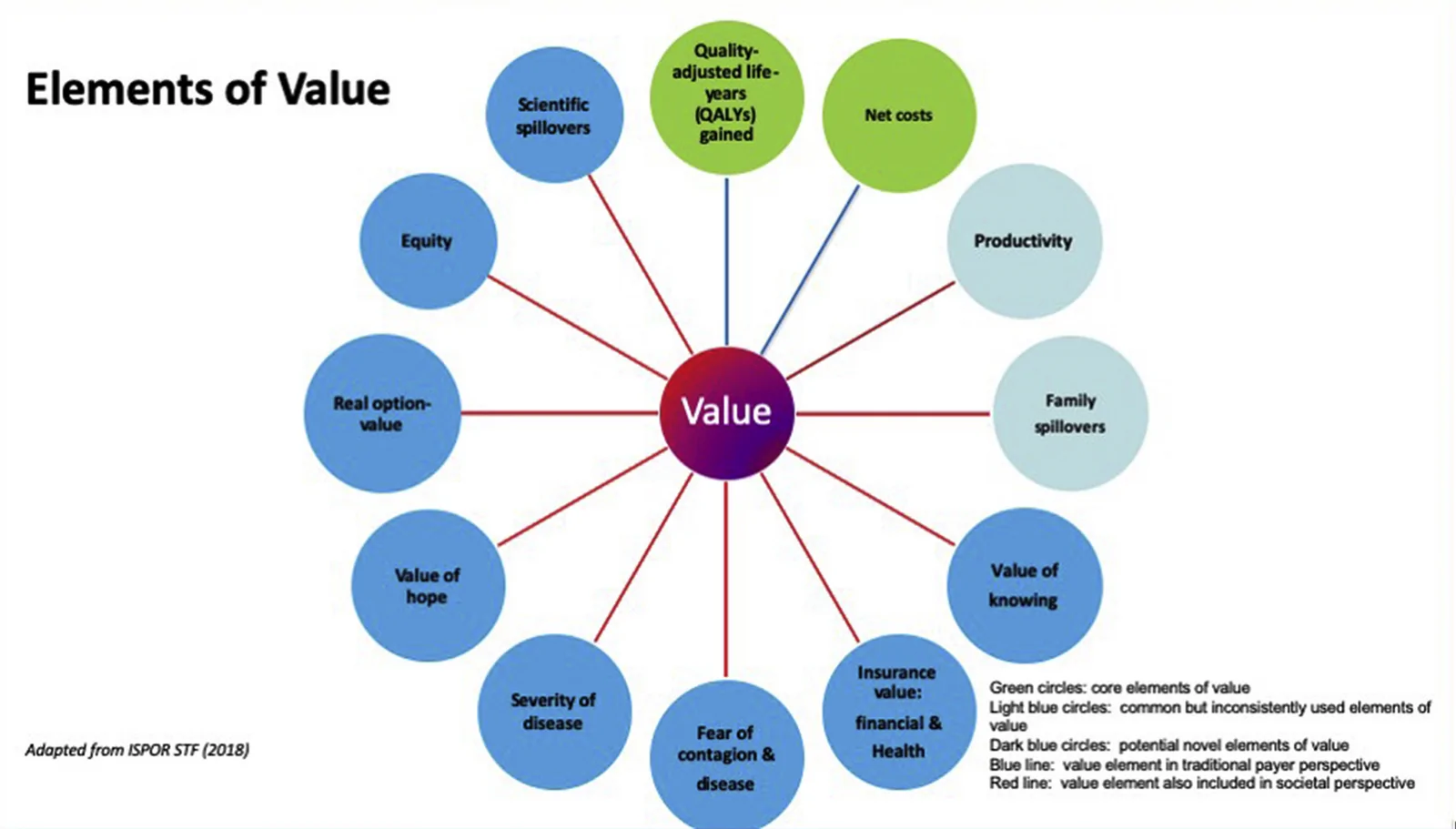
The value flower

The question remains what values should be taken into account, on what grounds, and how. One debated suggestion of a list of values to be considered is the so-called value flower presented by a task force from the International Society for Pharmacoeconomics and Outcomes Research (ISPOR) (Lakdawalla et al. 2018) (Fig. 1). ISPOR is by its own reckoning “the leading source for scientific conferences, peer-reviewed and MEDLINE^®^-indexed publications, good practices guidance, education, collaboration, and tools/resources in the HEOR [Health Economics and Outcomes Research] field”.^1^ Their task force work is generally influential for methodology and policy and this particular task force is no exception. Specifically, the implications for pharmaceutical pricing policy may be large and have not been discussed at length.

The task force emphasises that different suggestions for values could be considered depending on the remit and perspective of the relevant decision maker. The main concern being that conventional outcome measures such as the quality-adjusted life year (QALY) may not capture all relevant values produced by medical technologies. What values (and on what grounds) to take into account is a key consideration in publicly funded healthcare systems. This is so, for example, since the cost of new technologies such as pharmaceuticals will be handled within a dedicated budget for healthcare. Hence, the opportunity cost and potential displacement must be evaluated alongside the potential gains (Sculpher et al. 2017). The task force’s approach to value is founded in an economic perspective based on preferences where ”the “gross value” can be thought of what someone would be willing to pay for an economic good or intervention, whereas the “net value” subtracts the opportunity cost incurred to obtain that gross value” (Garrison et al. 2018, p. 124).

In this paper we explore the normative plausibility of these values and how they should be viewed from a value theoretical perspective. By doing so, we point to some ambiguities regarding the interpretation and implications of taking these values into account within a publicly funded healthcare system that relies on cost-effectiveness evidence in its remit to allocate healthcare resources in a way that balances efficiency (i.e., maximizing health) and distribution (i.e., equity concerns). If new elements are added to the cost-effectiveness assessment while the cost-effectiveness thresholds remain unchanged, any additional benefit may be “priced in” with all added value being allocated to producers and with potentially negative net effects for the health care system. In the article we focus on pharmaceuticals, and we argue against incorporating the novel suggested values as they stand into assessments of pharmaceuticals for reimbursement and allow them to affect pricing, at least in a publicly funded universal healthcare system of a European kind. To the very least, much more work is required if any of the petals (besides the ones already uncontroversial from the system’s point of view, i.e., QALYs, net costs, and severity) should be included in the assessment and pricing of pharmaceutical treatments.

The paper is structured as follows. We first make some assumptions indicating the perspective from which the analysis is made, then provide a brief overview of the value flower and of philosophical value theory. We then proceed to analytically discuss each of the petals of the value flower before we wrap up with general comments on practical implications and conclusions.

## Some preliminary assumptions

In this article, we consider a publicly funded healthcare system with a largely fixed budget over time. We also assume that considerations of efficiency (producing as much health as possible) are balanced against equity concerns (distributing health fairly). Moreover, besides more formal equality considerations, disallowing certain discriminatory criteria, we assume that equity concerns are mainly captured by using severity as a cost-effectiveness modifier, meaning that society’s willingness to pay for health gains increase the more severe condition that is targeted by the pharmaceutical in question. This is a practice found in Sweden, Norway, The Netherlands, and recently in England and Wales through NICE (Sandman and Juth 2024). Moreover, we confine ourselves to pharmaceutical treatments (sometimes in brief called “treatment(s)” since the policy implications of the task force´s suggestions are largest in publicly funded healthcare systems where reimbursement and pricing of pharmaceuticals are closely related to the cost-effectiveness assessment. That being said, we acknowledge that the argument we make may be, in some instances, applicable to treatments more generally, pharmaceutical or not, especially if they are produced and sold to the healthcare system on a market. However, this is not something we argue in this article.

### The value flower

While different versions of the value flower have been presented, (Garrison et al. 2017; Lakdawalla et al. 2018) we will use the petals from the most recent version (Neumann et al. 2022) (Fig. 1). However, when interpreting what the values are more specifically, we often refer to the more elaborated explanations present in the former versions (Garrison et al. 2017; Lakdawalla et al. 2018). Given the above assumptions, our focus is on the elements of value possibly complementing the petals of QALY, net cost and severity of disease, i.e., productivity, family spillovers, value of knowing, insurance value, fear of contagion and disease, value of hope, real option value, equity, and scientific spillover and the implications of adding those elements into the economic evaluation underlying decision making in a system accepting these assumptions.

### On value

Values come in different forms, and not all values are equal or even of the same kind. First, something can have value for its own sake, i.e., final value (Rønnow-Rasmussen 2015).^2^ Moreover, final value can be prudential, i.e., contribute to how well life is going for the person whose life it is, or moral, e.g., by, contributing to making the world better, for instance more just (which might coincide with making persons’ life better but not necessarily). Looking at theories of final prudential value, we find three basic approaches: hedonism, preferentialism, and perfectionism or objective-list theories (Parfit 1986). Hedonism claims that the value of a person’s life is determined by how that person experiences life, where positive experiences contribute to making life more valuable, and negative experiences lower the value of life. Preferentialism claims that the value of someone’s life is improved by having (certain of her) preferences satisfied and is made worse by frustrated preferences. Finally, objective-list theories, claim that certain specific aspects contribute to the value of a life, regardless of how they are experienced or whether people prefer them, like close relationships, autonomy, being active and contributing to the world etc. (Lin 2017).

Second, many events, aspects, situations, can have instrumental value in the sense that they contribute to something else that have final prudential or moral value, and they only get their value to the extent they contribute to such values. Hence, they do not constitute separate final values.

Looking at the value flower from these value theoretical perspectives, there seems to be values that have both a prudential and moral value from the perspective of healthcare. Benefiting patients in terms of net QALY-gain, even if clearly a prudential value, also seems to be at the heart of the moral rationale for having a healthcare system, voiced in terms of principles of beneficence and non-maleficence (Beauchamp and Childress 2019). The same might be true for other aspects like providing hope and adding to patients’ autonomy (e.g., through the value of knowing). Again, these are prudential values. However, in the value flower, there are petals that are moral rather than prudential values, e.g., the severity of the condition and equity. Before going into detail about the distinction and relationship between these two values, we could generally say, that both values seem to imply that we should be willing to sacrifice net benefit of prudential value, to give some priority to patients with more severe conditions, or to ensure equity. Hence, these are values that will put weights or constraints on a health (or value) maximizing approach.

The aim of the axiological analysis is not to criticise the flower for consisting of different kinds of values; that is and should be fine. Rather, the analysis, besides differentiating and specifying what kind of value each petal represents, allows us to point to some problems with some of the petals, for example, risks of double counting, risks of failing to notice that some values may also be disvalues, the need to establish a general causal or instrumental relationship between the value and some final value etc. In order to do so comprehensively for the entire flower, we will scrutinize all the petals (except, again, the uncontroversial ones).

### The petals of the value flower

In the following sections, we will go through the value petals one by one analysing (i) the concept, (ii) what kind of value it is, (iii) its application in a publicly funded health care system (described above), including (iv) its opportunity cost (with the exception of equity to be explained below). Each section ends with a conclusion for the specific petal. In the table below, we summarize the analysis (See Table 1).

**Table 1:** Summary of the analysis of the different petals of the value flower

Petal:	Type of value	Potential problems in implementation	Need for research and policy adjustments
Productivity	• Final value if meaningful activities are part of health outcomes (but then part of health measurements)	• Opportunity cost - for both health and productivity gains of other treatments	• Understand how productivity effects affect the different stakeholders of the healthcare system (especially patient groups with differently severe conditions)
	• Instrumental if focusing on the cost-aspect	• Risk of prioritizing treatments not in line with equity approach (in terms of severity considerations)	• Marginal productivity thresholds need adjustment
Family spillover	• Health effects for family = same value as for patients - final or central instrumental value	• Opportunity cost - for both patient and family health	• Adjustment of marginal productivity thresholds
		• Free-riding, if not taking severity of family impact into account	• Understand how different models for introducing family spillover will impact the healthcare system
		• Limitations - only one family member seems arbitrary	• Develop models for how to integrate and measure patient survival into family spillover
		• Measurement issues - especially related to prolonged survival	
Value of knowing	• Instrumental to subjective well-being and autonomy	• Opportunity cost - to patients’ health and knowledge	• Better understand how introducing this value will impact on the healthcare system
		• Context dependent whether having a positive or negative impact on the patient	• Adjustment of the marginal productivity thresholds
Value of hope	• Final value in terms of subjective well-being	• Opportunity cost to patients’ health and hope	• Adjustment of marginal productivity thresholds
		• Potential conflict with value of knowing	
	• Instrumental to well-being and autonomy	• Context dependent whether this has a positive or negative impact on the patient	• Better understand the impact on patients of taking value of hope into account
Insurance value	• Instrumental value in reducing fear and anxiety for negative health effects and financial risk	• Opportunity cost to patients’ health and to other treatments with insurance value	• Adjustment of marginal productivity thresholds
		• Introducing the value will imply potential price increase of the treatment in question - potentially making it more difficult to implement (and thereby increase the insurance)	
		• A healthcare system with universal coverage is a more optimal way to achieve this value	• Better understand the impact of the impact on patients of taking insurance value into account
Fear of contagion and disease	• Final value - since fear is a negative subjective experience	• Opportunity cost to patients’ health and to other treatments with positive effect on fear	• Better understand how introducing this value will impact on patients’ health and fear.
	• Instrumental negative/positive value that might impact general well-being, health and autonomy etc.	• Risk of strengthening irrational fears in population (in introducing and allowing higher costs for treatments for of objectively low risks)	
Real option value	• Instrumental value to future health effects	• Opportunity cost to present treatments for future (uncertain gains) that will be paid for when they arrive	• Adjustment of marginal productivity thresholds
		• Risk of double counting	• Better understand how such a value would impact on patients in the system
Equity	• Moral final or instrumental value	• Multifaceted concept - need for specification	• Further normative work is needed to describe which aspects of equity should be incorporated and if so, how they interact with other values of the system
		• Opportunity costs to patients’ health and to equity (in the form deemed important)	
		• Risk of overlap with severity	• Better understand how the outcome of such analysis would impact on the system if introduced
Scientific spillover	• Instrumental value to future health effects	• Opportunity cost to present treatments for uncertain future gains	• Adjustment of marginal productivity thresholds
		• Risk of double counting	
		• Ineffective way to support innovation	• Better understand how such a value would impact on patients in the system

## Productivity

### Concept

The task force defines productivity in terms of the increase in time being able to spend on working, in formal or informal labour markets or in household production (Brouwer 2018). The task force also acknowledges that there are both measuring issues and equity issues with this value. However, let us assume for the sake of the argument that we have no measuring difficulties and that we can assess all productivity effects (regardless of whether they result from formal or informal work etc.).

### Kind of value

As we interpret the task force suggestion, productivity value is a value of the same sort as QALYs and net costs: adding productivity value to a treatment will also add to its cost-effectiveness assessment and most likely imply a possibility to set a higher price for the treatment, most often since taking productivity effects into account reduce the net cost. According to that interpretation, productivity is obviously an instrumental value, in bringing down the net cost. However, following standard quality of life-metrics such as EQ-5D (Devlin and Brooks 2017), usual activities are also adding to patient’s health-related quality of life (HRQoL). Therefore, that aspect of increased productivity should (ideally) be captured by such metrics and could be viewed as a final value on an objective list-approach. Hence, here we focus on the cost-impact.

### Implications and opportunity cost

How would adding productivity value affect the distribution of value in the healthcare system? If an otherwise healthy person develops a temporary illness with low severity, this may temporarily affect the work ability of this person. Suppose that the condition’s impact on work ability is rather small (without treatment) – having to stay home from work a few days, perhaps have a somewhat reduced work capacity until it recovers spontaneously. Hence, any productivity gains of a treatment targeting conditions that returns the person to full work capacity will also be rather small, unless we aggregate the total productivity gain of a large population of such minor problems. This aggregative approach is, however, not something we generally find in the healthcare jurisdictions that use cost-effectiveness analyses to decide on reimbursement of pharmaceuticals (and will be ignored in the following).

At the other end of the severity scale, we might have a person with a very severe condition with a high impact on HRQoL and mortality, such a person is likely to have a very reduced work ability. However, only a substantial treatment effect is likely to have a substantial effect on the work ability. For example, a person suffering from end stage cancer, getting another treatment prolonging life with a few months, is not likely to return to work, even if he or she might be able to contribute somewhat more to the household chores.

In effect, the larger individual productivity gains will probably accrue to patients with conditions of moderate severity and where treatments have a fairly substantial effect. This in turn, implies that adding productivity gains will somewhat conflict with an emphasis on severity. It might therefore result in a different distributive pattern than what was intended by introducing severity as a priority setting criterion in the first place and allowed it to set the limits for acceptable approval thresholds.

What if we also consider the opportunity cost of taking productivity gains into account? Suppose there is a new treatment for a moderately severe condition with high impact on work ability introduced on the market. We assess that on top of the treatment reducing the severity of the condition, it will also bring increased productivity in the group, warranting a relatively higher price and thereby also a higher opportunity cost. In order for the total gain to the healthcare system to follow our distributive scheme, any displacement should affect conditions of lower severity or less cost-effective treatment for equally severe conditions. To ration treatment for patients with conditions of moderate or low severity is likely to have some negative productivity effects, unless we target patient populations which have less impact on productivity (older people, people with disabilities etc.) and this would be problematic from an equity perspective. If existing healthcare in the system is generally more cost-effective than the treatment to be implemented, more individuals will be affected by rationing than the ones benefited by the implemented treatment – hence the negative productivity effects might be substantial. Obviously, we would need to make a better assessment of this, since such negative productivity effects are highly relevant for whether we should include productivity effects or not. In doing so, we would need to identify the different stakeholders being affected, which besides patients, the healthcare system and the pharmaceutical industry, would also include employers. This leads us to the basic reason for why it would be important to include such effects.

By contributing to the work force, patients will add to the economy of the society and indirectly (through taxes) also to the healthcare system. Hence, from a societal perspective and perhaps also from a healthcare perspective, the net cost of the treatment could be lower. But this whole assumption rests on two different factors: there being a net productivity benefit from the implemented treatment, and the increased cost of taking productivity into account for the healthcare system is balanced by the increased tax return. If the displaced healthcare implies a large loss of productivity in the population, or the tax return is less than the increased cost to the healthcare system, this argument loses its force (Sculpher et al. 2017). If, on the other hand, it can be shown that there is a net benefit of resources to the healthcare system in taking productivity effects into account and where the potentially extra benefit to patients with moderate and lower severity is not associated with any displacement or cost to patients with higher severity – then adding this value could be acceptable. However, if we find the distributive scheme central, it seems the burden of proof should rest with those who argue for adding productivity values to the assessment.

In conclusion, it is not yet appropriate to incorporate productivity into pharmaceutical assessment because there is a risk of both conflicting with the basic distributive scheme if doing so does not de facto result in an improvement of equitable distribution of health or imply a balance over tax incomes over increased costs to the healthcare system.

## Family spillover

### Concept and kind of value

Indirect benefits to families involve the same kind of final prudential value that we refer to when taking the patients’ QALYs into account. Such benefits, e.g., for informal carers, have been discussed fairly extensively in the literature from different perspectives (Brouwer 2018; Brock 2003; Du Toit and Millum 2016; Gustavsson et al. 2023; Lippert-Rasmussen and Lauridsen 2010). It has been argued there is a prima facie argument for taking (at least) health effects to families or indirectly affected persons into account. Simply put, if we are interested in improving people’s health, why should we make a difference between direct health effects accruing to patients and indirect (but equally real) health effects accruing to other people (be they family, friends, work colleagues etc.)?

To simplify the following discussion, we will assume that we take one affected person per patient into account,^3^ and assess health effects (i.e., whatever outcome measure we take to be relevant for patients is also the relevant one for family members). So, what are the implications of introducing family spillover effects, related to aspects like severity and treatment effect?

### Implications and opportunity costs

First, for mild conditions, the family impact is likely to be low and thus, any treatment spillover effects are also likely to be small. For very severe conditions, the family impact is, on the other hand, likely to be higher. However, the relationship between patients’ severity and family spillover effects is not necessarily linear. More severely ill patients might have access to more formal healthcare, reducing the health impact of having to provide long term informal care. At the same time, conditions of more moderate severity, but chronic, might have a larger family impact. Patients with highly severe somatic conditions, might have less health impact on families than moderate to severe mental or psychiatric conditions etc. Following this, taking family spillover effects into account might not necessarily strengthen the distributive scheme we find central, but may draw in a different direction. Moreover, it has been difficult to find a way to model patient survival effects into family spillover, since a negative impact on the family is then prolonged and would hence worsen the family situation.

Second, and related to this, if health effects should be distributed in relation to the severity of patients, it should obviously do the same when considering spillover health effects on families. Once again, the health impact of a specific disease on the patient and on the family member is likely to be different. If this is the case, from a distributive perspective, we cannot just add the values of patient effects and family spillover effect and apply the approval threshold following from the severity of the patient’s condition, since then the family member would “piggy-back” on this condition getting a higher priority than what is warranted (if the patient has a higher severity than the family member). The higher cost and thereby also opportunity cost of such a practice, would also risk displacing healthcare for more prioritised treatments in the system (even if we try to ration less prioritized healthcare based on the severity and cost-effectiveness of the situation for the patient). Hence, we would need to distinguish between family severity and patient severity, and also between the patient effect and the extra family effect – and then apply different cost-effectiveness thresholds to the different effects. This is not impossible, even if it will make the HTA more complex.

However, if we start taking family spillover effects into account for new treatments to be implemented into the system, we also need to take into account the family spillover effects lost through displaced healthcare, i.e., the opportunity costs. Since the marginal productivity of the healthcare system would change taking into account family spillover, this opportunity costs will then be higher than when only taking patient effects into account. Generally, taking family spillover into account would imply that the current system is much more effective in producing health than if we only take patient effects into account and hence the marginal productivity level of the system would be higher. Once again, the net benefit of family spillover might be negative, if taking this into account in relation to a specific treatment result in rationing other healthcare treatments with greater (already existing) family spillover.

In conclusion, it is not yet appropriate to incorporate family spillover into pharmaceutical assessment because unresolved challenges of avoiding both negative net health benefits, and problematic equity effects.

## Value of knowing

### Concept

In a White paper on the value of diagnostics and prognosis, representatives of the ISPOR task force develop the concept of “value of knowing” (Garrison et al. 2016). While it is unclear whether the value of knowing is supposed to include some of the other suggested values (e.g., insurance value), we will understand “the value of knowing” in terms of reduction of uncertainty. Furthermore, while the White paper focuses on complementary diagnostics, we will analyse the value of reduced uncertainty from a more general perspective. There are several different ways in which the value of knowing comes about. In the White paper, this is referred to as “additional value from knowing a technology is more likely to work” (Garrison et al. 2016, p. 5). However, they also include the value of understanding the prognosis for the patient, with as well as without treatment. Moreover, this knowledge is likely to come from different diagnostic measures and people may value a predictive test even if it does not have any immediate treatment consequences.

As observed in the White paper, reduced uncertainty about which patient will respond to which treatments or about the future prognosis of the patient will enable better adapted treatment choices or stimulate behavioural changes to reduce projected risks. Hence, thus related to actual health effects. Such predictive or prognostic information might also influence costs, e.g., saving unnecessary costs for less-than-optimal treatment. However, as noted, collecting this information comes at a cost and might also incur new costs, e.g., having to deal with incidental findings. In any case, there are obviously instrumental values in reducing uncertainty to both sides of the ICER and should be taken into account in the traditional CEA. The White paper also emphasises the value of epidemiological knowledge for R&D investments and planning of healthcare expenditure to address healthcare needs.

However, the authors also suggest that there is another value of reduced uncertainty, unrelated to health outcomes. They suggest that patients value a reduction to uncertainty independently of the expected health outcomes and that value may be explained by *psychological benefits* to risk-averse patients, by positive effects on patients’ *planning* (Garrison et al. 2016), and potentially even by ‘bringing a peace of mind’ to non-patients by the mere availability of new effective treatments (Lakdawalla et al. 2018 p. 135; Neumann et al. 2022 p. 559). Moreover, they note that reduced uncertainty may encourage favourable *behavioural adjustments*. Finally, they suggest that the value of reduction of uncertainty can also be spelled out in terms of the *well-being* generated from more information (due to less ambiguity and the additional information working as reassurance for patients).

### Kind of value

All these descriptions indicate that we are considering a prudential value. It is also clear that this value is not final, i.e., it is a form of instrumental value to achieve aspects here described as psychological benefit, well-being, better planning, changing risk-behaviour, behavioural changes. Furthermore, the instrumental relation between reduced uncertainty and these values (and we will get back to whether they in turn are instrumental or final) is not one of necessity, but a probabilistic relation. That is, it is not necessarily the case that if the patient gets information that reduces uncertainty, s/he will experience these potential benefits. Hence, over the relevant consideration is to what extent it is likely that it generally has such an instrumental relation, and then also, to what extent reduced uncertainty might actually be instrumental to negative values. So, let us look at these different values.

### Implications and opportunity cost

Let us start with psychological benefit or, what we will interpret as, *experiential* well-being from reduced uncertainty. First, this is a strong candidate for having final value, in hedonism definitely so, but also on an objective-list theory, i.e., a value pluralist theory saying that a number of items have prudential value for a person (see above). It is not in preferentialism, but many (if not all) people will to some extent prefer having experiential well-being (it is difficult to imagine a human person (living a good life) having no preferences at all concerning this). Obviously, reduced uncertainty might reduce anxiety, improving experiential well-being. However, we might as easily imagine situations when it will increase anxiety, for example diagnostics providing information about a very poor prognosis, or that no available treatment will have any chance of succeeding in alleviating the condition etc.

Sticking to the simpler instrumental relationship between reduced uncertainty and experiential well-being, it will be an empirical matter whether a certain diagnostic test, on balance, will lead to more, or less experiential well-being among the patients concerned (an effect that might be multiplied if we also consider indirect effects on family etc.). Spending resources on reducing uncertainty will also have an opportunity cost, with potentially negative effects for other patients in terms of reduced access to care. However, this opportunity cost might also have implications for other patients’ certainty or uncertainty, with the resulting instrumental effects on experiential well-being. This will probably be difficult to assess, but one case could be if there is limited analytic capacity of diagnostic tests due to shortage of staff at the lab and where introducing a new test that (let us say) lead to, on balance, experiential well-being for the patient population, but at the expense of longer waiting times for receiving the results for other patients’ diagnostics and hence prolonged uncertainty.

Let us now turn to planning and changing behaviour. Depending on how we plan and how our behaviour changes, the resulting effects may be instrumentally valuable. But a reduction of uncertainty may more generally also influence autonomy which, in turn, is a candidate for final value. However, this effect may not necessarily be positive. Consider the following case:

Liza is suffering from cancer. She has received curative treatment, but there remains a risk of remission, and that risk varies depending on her genetic make-up. Depending on which risk-type she is (A, B or C), the remission will have more or less negative effects. For A, with a high risk of remission, there is no available treatment, and she will die within a couple of months of remission. For B, with a moderate risk of remission, there is a highly effective treatment with which she is likely to be cured. For C, there is almost no risk of remission.

Information about what risk-type Liza belongs to may impact on her exercise of autonomy. Suppose that Liza is in her twenties and is considering pursuing medical school to later practice medicine as a specialist. However, doing this will take much time from friends and other interests she cares about, which she considers to be a sacrifice. Suppose that she finds out that she is in risk-type A, then she would not be willing to make that sacrifice. Initially, this may be thought of as having a positive impact on her exercise of autonomy. But, getting to know that she is in risk-type A might also make her fall into a deep depression where she would lose all interest in spending time with her friends as well as her other interests. So, more information may not necessarily have a positive impact on a patient’s autonomy. How a reduction of uncertainty will impact the autonomy of a patient is individual and context dependent. While one could argue that more information has a stronger relationship to autonomy, than to experiential well-being, it is still an empirical question how it will affect a patient population and whether it, on balance, will have a positive impact on autonomy. Once again, spending resources on reducing uncertainty for a specific group, might have an opportunity cost in terms of increased uncertainty, or more directly decreased autonomy for another patient population, e.g., in reducing available care options etc.

In conclusion, it is not yet appropriate to incorporate the value of knowing into pharmaceutical assessment because it is difficult to find support for a uniform and positive effect of this value. Also, it is also uncertain whether allowing the value of knowing to incur a greater opportunity cost will result in a net benefit or loss of the values related to knowing.

## Value of hope

### Concept and kind of value

At first glance, it may seem obvious that the value of hope should be taken into account in decision-making about pharmaceutical pricing. This is also expressed in Lakdawalla et al. 2018: “The basic idea underlying the value of hope is intuitively plausible: many severely ill patients may be willing to trade off some survival (e.g., undertaking a risky procedure) for a chance at a “cure” — even if only for a small probability of that much longer expected survival or cure” (p. 136). However, how the notion of hope should be understood more specifically, and how it could serve as a basis for decision making in pharmaceutical funding is less clear.

While there is a small but growing literature on the notion of hope in bioethics (Ruddick 1999; Blumenthal-Barby and Ubel (2018; McMillan et al. 2014; Eijkholt (2020), we believe that hope can be understood in terms of the following three conditions for the concept of hope: “…hoping is just having the proper belief and desire in conjunction with being engaged to some degree in mental imaging”(Bovens 1999 p. 674). Let us briefly elaborate on each of these conditions. First, the belief must be uncertain. It does not make sense to say that I hope for something that I am absolutely certain about. Neither does it make sense to say that I hope for events that are in some sense impossible to realize. Second, the strength of the desire will determine to what extent I am better off would the desired outcome occur as well as the extent to which I am worse of would the desired outcome not occur. This can, for example, be cashed out in hedonistic terms where the experienced (dis)pleasure would determine my decrease or increase in prudential value, whereas preferentialism would evaluate the realization of the outcome in terms of preference fulfilment. Third, the mental imaging is related to the pleasurable experience that I may have since it seems to be part and parcel of the notion of hope that we “In hoping for something, [I] tend to fill in the contours in the brightest colors” (Bovens 1999, p. 670). Note that whatever hedonistic gains this colouring may result in, they must be weighed against the hedonistic losses would the things I hope for not be realized (which could of course potentially outweigh the gains). So, even if one thinks that value of hope should be taken into account in priority-setting, it is certainly not clear that it would actually imply accepting higher costs.

### Implications and opportunity cost

Let us now say something about the instrumental value of hope. Hope can be valuable in terms of “the enabling function” (Bovens 1999, p. 670) which means that my hoping for the preferred outcome may change my behaviour in certain respects. I might start undertaking certain actions that may (or may not) increase (or decrease) my chances of obtaining the preferred outcome. In relation to treatment, my hope for a longer life or better health may, for example, motivate me to strictly follow a treatment plan (or perhaps even start the treatment in the first place). However, note that the impact of hope on behaviour may not be positive on a patient (given that the disvalue is greater than the value coming from the feeling of hope). For example, suppose that the aforementioned Liza pursues lengthy medical school studies (in favour of spending more time with her friends and family) because she has some hope of being cured. Suppose further that after some time she finds out that she is in the first risk type (and hence will die shortly after remission). In such a case, Liza may regret her previous choice and be overall worse off because of that choice which was influenced by the hope she felt. In such a scenario, hope would amount to more disvalue than value.

Let us now turn to some worries about the implications of introducing the value of hope in this context. First, recall that hope is partly constituted by the “proper belief”, that is, a belief that has at least some degree of uncertainty. Accordingly, there is a tension between, on the one hand, value of hope, and on the other hand, the value of knowing. For the value of hope to be relevant in the context of pharmaceutical pricing, there must be some uncertainty. Consider a patient group consisting of 100 patients. They all suffer from condition Y. The current treatment option for Y is treatment T which is effective in 25% of the cases. However, since there is uncertainty around which 25 out of the 100 patients will benefit from T one has to treat all 100 patients. It seems reasonable to say that every individual patient in the group hopes to be among the 25 who are cured. Therefore, we should take this value into account in addition to whatever the QALY-gains are. However, suppose next that a new medical technology is introduced on the market. Suppose that this is a diagnostic measure that can with 100% certainty identify which 25 out of the 100 we should treat. In such a scenario, the value of hope disappears. The situation is made worse. This seems strange.

One may correctly point out that while it is true that the situation is now made worse in one respect (less (or no) value of hope), it is much better in other respects such as better cost-effectiveness (due to less over-treatment and lower costs). And therefore, one might suggest, although there is some negative value in the loss of hope, this is outweighed by the health effects.

The problem with this reply is that it still seems counterintuitive that an increase in certainty (with regard to effects and side effects) together with lower costs would in any way result in a situation counting against funding the new diagnostic measure.

Second, part of the support for the value of hope is, according to Neumann et al. 2022; empirical support for the fact that people value hope. This is expressed in terms of increased willingness to pay for a “treatment that provides a sizable chance of “cure””. But this is far from the only interpretation of the empirical data. Another interpretation of the results would be that what people ascribe value to in these studies is not *hope* but *cure*. People simply value the increased chance of cure more than “mere” expected health benefits. Therefore, to ascribe value to hope seems to be at least partly to confuse the value of hope with other values such as the value of cure. Also, perhaps needless to say at this point, adding this value would have an opportunity cost. If the value of hope implies paying more for some drugs at the expense of already existing treatment in the system, and this de-implementation or watering down of certain treatments, affects a large number of patients, reducing their hope – we might get a net loss of hope.

In conclusion, it is not yet appropriate to incorporate the value of hope into pharmaceutical assessment because while the value of hope may be important in assessing new treatments, its inclusion in funding decisions is may introduce, for instance, additional uncertainty and opportunity costs. Therefore, even if hope has value, it does not straightforwardly justify higher spending without careful consideration of trade-offs and overall health outcomes.

## Insurance value

### Concept and kind of value

Insurance value refers to the willingness to pay for access to something that one does not necessarily use. In the context of healthcare more specifically, the concept has two components. First, insurance value refers to the concept that even though some diseases do not pose a risk to everyone, in general, the number of consumers willing to pay for a medical technology exceeds the number who will ever need it (Lakdawalla et al. 2018). This reflects the “physical risk protection” value created by a new technology. Second, it can be argued that new medical technologies make financial insurance policies more useful as greater options for medical care expand the possibilities for insuring against illness; if there is a novel treatment for a condition that previously could not be treated, there is now possibility to insure for the condition in question (Lakdawalla et al. 2018). This is sometimes called “financial risk protection” for the healthy. Together, these two components—physical risk protection and financial risk protection—constitute the “insurance value” of medical technology, and it is claimed that this value is most relevant to take into account when it concerns severe diseases (Lakdawalla et al. 2018). This petal has an overlap with the values of hope and the value of reducing fear of contagion and disease. Obviously, this has an instrumental value in terms of risk protection.

### Implication and opportunity cost

A healthcare system with universal coverage is arguably the best insurance against such risks. Such a system, which tries to incorporate new treatments for severe illnesses and diseases will handle the above problems to the extent they take aspects like severity (and for contagious diseases also spread) into account. However, there seems to be a kind of paradox if allowing the insurance value to also impact on the pricing of the intervention, especially in a publicly funded healthcare system. If the price set by reference to the values of severity and QALY-gain of the intervention should also incorporate some additional insurance value, the price will be higher. With a higher price, the opportunity cost will increase, likely making it more difficult to incorporate the intervention into the healthcare system, and therefore in essence reducing the insurance value related to the intervention. Now, it might not be about increasing the price, but rather a factor that should have us accept cost-effectiveness levels not in line with standard cost-effectiveness thresholds. However, such an approach will have the same implications for the healthcare system, but then more indirectly, since this will imply that we accept a higher opportunity cost for such a drug than what is motivated by the central factors of severity and QALY-gain. The healthcare interventions, displaced as a result, could well be interventions that are more effective in producing health (in relation to severity) – which in turn might, on balance, reduce the insurance value of the healthcare system. Especially, if the displaced care involves making basic care for large portions of the populations less available.

In conclusion, it is not yet appropriate to incorporate insurance value into pharmaceutical assessment because in a publicly funded healthcare system with universal coverage, it is difficult to motivate a role for the petal of insurance value.

## Fear of contagion and disease

### Concept

Fear of contagion and disease refers to the disvalue of the anxiety associated with the risk of future spread. As Lakdawalla et al. 2018 notes, the benefits of preventing people from actually being infected are typically included in value assessments, while the fear associated with the spread of disease is not. Reducing the anxiety associated with the risk of future spread may be valuable to the society, even if the expected number of cases prevented is low. The authors argue that in contrast to other factors, fear plagues all consumers who might potentially be exposed to the disease, not just those who are sick or are actually exposed.

### Kind of value

Now, fear has a negative experiential (final) value and will, all things equal, lower a person’s QoL. Fear might also have negative instrumental value in restricting people’s action unnecessarily in a way that might reduce QoL but potentially also a positive instrumental value in helping us avoid things that might threaten us to the extent of also lowering our QoL and even killing us. Now, we know from risk research that there is not a direct correspondence between the risks we fear in situations (i.e., our risk attitudes) and how risky these situations are more objectively to us (Weber 2001). We might have a strong fear of risks that are very unlikely to be realized, and almost no fear at all for risks that are serious and much more commonly will be realized (e.g., flying vs. driving a car). However, even accepting this, it could be argued that it is still valuable to reduce also potentially irrational fears, due to the negative experiential value of fear.

### Implications and opportunity cost

Generally, it seems as if effective treatment that will actually reduce the objective risk of contagion and disease is the best cure for such fears, and to the extent it is effective, this will also show in the cost-effectiveness assessment. So, in which situations could it potentially be motivated to also include the further value of reducing fear? As indicated in the quotation, this might include situations when the objective risk is low for contagion or for contracting the disease, but it still sparks fear in people. In such cases, the effect side of the cost-effectiveness assessment might not have that much to show for itself, and still the treatment (e.g., vaccination) might need to be distributed to a large population to reduce fear. If such treatment for this reason will have difficulty making standard cost-effectiveness thresholds, we might see reason to add other values to the equation to increase the potential beneficial effect. Take Ebola as an example. In societies where there is a more substantial risk of the spread of Ebola, this would not seem to be a problem. An outbreak of Ebola would have devastating effects for such a society, and hence the potential beneficial effect in avoiding this would not be very problematic to catch and give a substantial value in the cost-effectiveness assessment (especially if we also include further social externalities). On the other hand, in countries like Sweden, even if there, for some reason would be an increase in the spread of fear of Ebola, preventive treatment does not seem warranted, given the objectively low risk of this happening. Furthermore, this would be an unmotivated opportunity cost to the healthcare system, impacting on more beneficial treatment. A better way to handle such fear in society would probably be, for instance, information campaigns about the risk of contracting Ebola. In other words, introducing the petal of fear of contagion and disease, will risk having us pay for irrational fears that we should find better ways to mitigate. One crucial role in this could be a universally covering healthcare system, where we trust that the system will take care of us, to the extent possible.

However, let us assume we allowed this to be taken into account, how would this generally affect fear of contagion and disease in the healthcare system? If the opportunity cost of taking this aspect into account will displace other healthcare interventions, this might obviously increase fear of contagion and disease in the system. To the extent this affects a greater share of patients, this might be, on balance, worse for the level of fear of contagion and disease. This should be factored into this equation if deciding to take the petal into account. Of course, the fact that contagious diseases spread should be factored into standard cost-effectiveness assessment.

In conclusion, it is not yet appropriate to incorporate the fear of contagion and disease into pharmaceutical assessment because it might have us pay for irrational fears, better mitigated in other ways. Moreover, if the cost of reducing such fears become too large, the total net fear mitigating effect can be negative due to the opportunity cost for existing treatment in the healthcare system.

## Real option value

### Concept and kind of value

As noted by Lakdawalla et al. 2018, patients face uncertainty about when and how future advances in medicine will occur. Thus, extending life provides patients with an option to enjoy these uncertain future benefits. This “real option value” is generated when a health technology that extends life creates opportunities for the patient to benefit from other future advances in medicine. According to the authors, if a patient had to choose between two treatments offering the same expected QALY gain, the one that offers the greater life expectancy may be preferred as this provides a greater chance to benefit from access to future scientific and clinical advances.

As this petal refers to real option value it seems to focus on the potential for future improvements that can be achieved if medicine advances further during the life-extending period, rather than just some kind of “peace of mind” associated with healthcare and treatments being available should one need them in the future - sometimes referred to as option value more generally. Hence, we interpret this value to be of the same sort as QALYs, with life-extending treatments potentially leading to more expected future QALYs than quality-of-life enhancing treatments. Real option value is obviously thus an instrumental value, in potentially enabling experiencing future health. Hence, it will only have value, to the extent it actually fulfils this goal.

### Implications and opportunity cost

The argument by Lakdawalla et al. 2018 seems to be that standard cost-effectiveness analysis does not account properly for future potential benefits of life-prolonging pharmaceuticals. They suggest that a life prospect of 10 years with a quality of life of 0.25 is preferred over a life prospect of 5 years with a quality of life of 0.5, and that this preference, if it exists, is not captured in standard cost-per-QALY analysis. Considering real option value and our numerical example above, one may then argue that the preference for the prospect with a longer life expectancy is a matter of estimation. Assume there is a 0.5 probability that a new treatment becomes available after 5 years, and that this treatment improves quality of life from 0.25 to 0.75. The expected QALYs with this prospect is now 3.75^4^ rather than 2.5 and may explain the preference of this prospect over the one with a shorter life expectancy. It is ultimately an empirical question to establish if this is the case, however. If such a value exists, it is still not clear how it should be applied in a reimbursement context within a publicly funded healthcare system. Although empirically challenging it is conceptually uncomplicated to estimate potential gains with a particular probability and incorporate this as an expected QALY gain in the analysis. Such an approach may, however, have implications for distribution of health and opportunity costs if it also forms the basis for pharmaceutical price setting. If the expected QALY-gain of 1.25 is priced in when reimbursing the drug, current patients will pay the opportunity cost in terms of forgone health now (with certainty) for an uncertain future health gain of other patients. The distributional consequences of such an approach are difficult to assess; one must know the disease severity of patients forgoing and gaining health, today and in the future. It seems reasonable to believe that the gainers, both short and long term, will be patients with diseases impacting life-expectancy over patients with diseases primarily impacting HRQoL. In the short term expected QALY gains will simply be higher for life-prolonging treatments. In the long term it is likely that such an approach would also provide incentives for R&D on life-prolonging treatments compared to HRQoL-enhancing treatments. This need not conflict with the severity principle if life-expectancy is deemed more important for disease severity than HRQoL, but we believe this is far from always the case. In terms of opportunity costs, it would clearly matter which patients would bear the increased costs of paying for expected future health effects. If these are patients with diseases also impacting life-expectancy this will be a zero-sum game for the healthcare sector (as the “losers” will also lose the real option value) and the gainers will be the drug companies with life-prolonging drugs. Another conceptual issue with the incorporation of option values into the evaluation and pricing of new drugs is the fact that if a new treatment actually becomes available to achieve the 1.25 estimated QALY gain in the example above, how should the value of this drug be assessed if this expected value has already been paid for when reimbursing the drug that generated the real option value of 1.25?

In conclusion, it is not yet appropriate to incorporate real option value into pharmaceutical assessment because incorporating this element of value into cost-effectiveness analysis, and ultimately into drug pricing leads to, for instance, undervaluing HRQoL.

## Equity

### Concept and kind of value

When the task force discusses equity, they acknowledge the lack of a precise definition and refer both to motives of altruism and equal health outcomes across poor and rich people, as well as equal health outcomes across more or less healthy people. They also refer to concept of “rarity” and “unmet need” as potential considerations (Lakdawalla et al. 2018). In essence, we are not given much guidance as to how we should understand this concept, despite a rich philosophical literature on concepts of equity (Hirose 2015).

Equity is one of the terms used to refer to distributional issues within healthcare and society at large: it is not only important whether introducing new pharmaceuticals result in net benefit or net loss of the value or values in question; it is also important which patient groups are benefited and which patient groups carries the losses (which could be said to be a moral value). In this sense, equity is akin to concepts like justice and fairness (and equality, see below). If equity is to be taken into consideration, several difficult issues need to be addressed value-wise. 

### Implications and opportunity cost

First, there are a number of substantial views on what kind of equity that matters (and why) (Hirose 2015). One is prioritarianism, according to which benefiting people matters more the worse off they are. The idea is that value itself has diminishing marginal moral importance: the more you already have, the less additional value is worth (and conversely). This prioritarian notion is akin to, or very closely related to, severity as a priority setting criterion in combination with cost-effectiveness, since the very idea of severity is that we should be willing to spend more resources on those worse off in terms of health (or whatever currency one is labouring with) (Juth et al. 2025). Hence, equity in the value flower cannot be considerations of severity – otherwise they would be the same petal split into two with different names.

A more interesting suggestion on how equity should be understood in this context is in line with so called telic egalitarianism (henceforth egalitarianism), the notion that it is bad in itself if some people are worse off than others. From a value perspective, this would be an intriguing suggestion since it is a clearly separate notion of final value. No double counting or conflating instrumental and final value would be involved. Moreover, it is by definition a relational value, since it ascribes value to people being on the same level of health and negative value to people being at different levels of health.

However, intriguing as it is, egalitarianism has issues of its own that needs to be addressed if involved as a value in drug pricing. To start with, it takes substantial normative argument to make credible that (in-)equality has (dis-)value in itself. It has been argued that prioritarianism caters for such equity-based concerns while avoiding several of the shortcomings of egalitarianism, e.g., the levelling down argument (Parfit 2000). Moreover, egalitarianism is much more complicated than first meets the eye: there are at least nine accounts of what kind of inequality that is bad with nine different measurements tied to them (Temkin 1993). Here, substantial normative argumentation is also needed to sort out what kind of equality that matters and why (Juth et al. 2025).

Second, as pointed out by the task force (Garrison et al. 2018), the issue of currency needs to be addressed: in terms of what outcome should inequity be understood? Since we are dealing with healthcare, a straightforward suggestion would be to say that we should focus on equity with regard to the same currency we use to measure benefits, i.e., some sort of notion of quality adjusted life years where quality is specified in terms of health-related quality of life (for instance, as measured by EQ-5D). However, broader currencies have been proposed as relevant also in healthcare (Crisp 2002).

Third, equity could (also) be understood in terms of formal equality: that equal cases should be treated equally. Then it is not a value, but a basic norm of rationality that should be respected regardless of which more substantial idea of equity that is proposed. Although formal, this norm plausibly has some practical implications (Sandman 2023). For instance, it may be the ground of compensating orphan drugs for the regularly high costs per patient they entail (Juth et al. 2021). However, again, then we are not dealing with a value but with a justice-based constraint.

In summary, to introduce equity as a separate and general value-petal would take considerable normative work. Such work has to include different conceptions of equity’s implications on the healthcare system. Actually, part of the normative evaluation would include these implications, since the implications are relevant to evaluate the normative reasonableness of the system, including the opportunity costs of catering for equity-based considerations (Juth et al. 2021). Hence, a great deal of theoretical groundwork is needed before we could even start to consider the practical implications of inviting equity into the value flower.

In conclusion, it is not yet appropriate to incorporate equity in general into pharmaceutical assessment because much work is needed in order to clarify what it means as something separate from already established equity-based concerns, such as severity.

## Scientific spillovers

### Concept

The idea behind the value of scientific spillovers is that certain health innovations provide the basis for further health innovations that, in the end, benefit patients and that this is not reflected in the price of the original innovations. In such cases, scientific spillovers are unrewarded positive external effects of health innovations. The idea is that value-based pricing should reflect such positive effects. A common example is when a novel drug identifies a previously unknown causal mechanism that sparks the innovation of new drugs using that mechanism (or mechanisms relevantly similar). So, rewarding scientific spillovers would only be “[r]elevant when technology identifies a new mechanism of action or mode of delivery” (Lakdawalla et al. 2018, p 134).

It is somewhat unclear if the idea is that scientific spillovers should be rewarded (as a matter of desert) or encouraged (by creating incentives to future fruitful innovations). As it is formulated, we interpret it as the latter (for the sake of “future generations of patients” (Lakdawalla et al. 2018, p 137). Also, to import considerations of desert into pharmaceutical pricing would be a radically new suggestion. It could then seem odd to encourage something that has already happened (since we can only pick out which innovations that have led to further innovations post fact). However, the thought, we suppose, is that rewarding scientific spillover would be part of a system that, in general, encourages fruitful innovations.

### Kind of value

What kind of value would then scientific spillovers be? It is obviously an instrumental value. Medical technologies or treatments are not valuable for their own sake, novel or not, but what we can do with them is what make them valuable. So, scientific spillovers are valuable to the extent they give rise to further treatments for the benefits of patients. If one thinks that knowledge is a final value (although we are sceptical about that), then innovations is a means of achieving that too.

### Implications and opportunity cost

Here the risk of double counting is evident: if we reimburse companies for scientific spillovers, they first get reimbursed for the potential of future treatments and then reimbursed again for the patient benefits (and/or knowledge) that always is reimbursed when the potential is realized and the new treatments comes on the market. Moreover, it encourages companies to play up the scientific value of new products, which may result in misleading information about novel drugs.

Also, even if one agrees that scientific spillovers should be economically encouraged, it remains an open question whether, and to what extent, this should be done by pricing of the innovative product in question. There are certainly other ways to encourage investments into fruitful scientific innovation, such as private and public research funding directed towards basic research. This has already long been happening and it requires further substantial argument to make credible that burdening the health care system with doing this through drug pricing would work and would work better than other ways of achieving the same end. Also, the general point of opportunity cost is applicable here too: if we spend more resources on rewarding scientific spillovers, these resources cannot at the same time be used elsewhere.

Actually, the value of scientific spillovers is a double-edged sword, since it could also be used to argue in favour of reducing reimbursement for health technology, e.g., pharmaceuticals. If a pharmaceutical is fruitfully innovative so it can be used as a vehicle for further drug development, then the pharmaceutical industry profits more from it in the long run. Because of patent rules, a likely candidate for such profitable returns is the very company that develops the original innovative drug. Then they can foresee more revenue in the future and should therefore be paid less for innovative pharmaceuticals.

In conclusion, it is not yet appropriate to incorporate scientific spillover into pharmaceutical assessment because of a number of principled and practical problems, for instance risks of double counting.

## Conclusions and implications for practice

In this article, we have scrutinized the petals of ISPOR’s value flower from the point of view of value theory and their practical implications for a public healthcare system of a European kind, that factors in considerations of cost-effectiveness and justice (primarily in terms of severity) to start with. We have argued that much work is required if any of the petals (besides the ones already uncontroversial from the system’s point of view, i.e., QALYs, net costs, and severity) should be included in the assessment and pricing of pharmaceutical treatments.

All the different petals obviously represent some form of value, following value theory, even if we have shown that this value is often more complex both conceptually and in terms of how to understand the petal in terms of value aspects. Moreover, we have also indicated that these petals do not necessarily have one-directional values, meaning that they can have both positive and negative value. Even acknowledging the value status of the petals, does not necessarily imply they should be incorporated into and allowed to influence pharmaceutical pricing. First, if incorporated into pricing models or assessment of price, more analytical and empirical work is needed to understand and normatively discuss how the values should be balanced against established values. Additional work is also needed to assess the opportunity costs of incorporating them and to determine the extent to which such costs might be acceptable. Second, incorporating such values without re-assessing the marginal productivity and accepted cost-effectiveness thresholds of the system, will risk resulting in a value transfer from the healthcare system (i.e., patients) to the pharmaceutical industry. Third, as we have shown in relation to the petals on insurance value and fear of contagion and spread, universal health coverage might be a better instrument to achieve these values or, in the case of irrational fear, public information might be alternative interventions to consider. More generally, it is important to emphasize, that when considering values we want to achieve with our healthcare systems, there are more ways than to incorporate them into health technology assessment of individual therapies. This is of particular importance for a publicly funded healthcare system that cares about the distribution of values across its patients as well as the distribution of values across patients and the pharmaceutical industry. The latter is not a trivial aspect. Although it is well recognized that pharmaceutical pricing, and thus the cost-effectiveness assessment, is endogenous to the design of the system (i.e., what values to account for) (Klockhoff et al. 2026; Jena and Philipson 2013), the task force has been relatively silent on this issue. The analysis provided in this paper is an attempt to open such a discussion.

In conclusion, at this time and given the perspective of a publicly funded universal healthcare system where considerations of cost-effectiveness and severity are established considerations, we remain sceptical about incorporating the suggested values as they stand into assessments of pharmaceuticals for reimbursement.

## Data Availability

Not applicable. This study is theoretical and does not involve data or material gathered from persons or animals. Hence there is no dataset to be made available. Any questions about the study can be directed to niklas.juth@uu.se.

## Footnotes

1 ISPOR 2026: https://www.ispor.org/about/our-society (accessed 2026-06-22).

2 There is a further distinction between final and intrinsic values: something has intrinsic value in virtue of the value bearer’s internal features alone, while final values may also have value for its own sake in virtue of relational properties. This distinction is not important in this text and will therefore be left aside in the following.

3 Note that this is a major simplification, since the question of which relations qualify as relevant is itself one of the issues that must be addressed for this kind of consideration to play a role in priority setting.

4 ([0.25*10]*0.5+[0.75*5 + 0.25*5]*0.5)
